# G-protein coupled receptor expression patterns delineate medulloblastoma subgroups

**DOI:** 10.1186/2051-5960-1-66

**Published:** 2013-10-10

**Authors:** Kelsey L Whittier, Erin A Boese, Katherine N Gibson-Corley, Patricia A Kirby, Benjamin W Darbro, Qining Qian, Wendy J Ingram, Thomas Robertson, Marc Remke, Michael D Taylor, M Sue O’Dorisio

**Affiliations:** 1 Department of Pediatrics, Carver College of Medicine, University of Iowa, Iowa City, IA 2524 JCP, USA; 2 Neuroscience Graduate Program, Carver College of Medicine, University of Iowa, Iowa City, IA, USA; 3 Holden Comprehensive Cancer Center, Carver College of Medicine, University of Iowa, Iowa City, IA, USA; 4 Department of Pathology, Carver College of Medicine, University of Iowa, 200 Hawkins Drive, Iowa City, IA, USA; 5 Queensland Children’s Tumour Bank, Queensland Children’s Medical Research Institute, The University of Queensland, Brisbane, QLD, Australia; 6 Pathology Queensland, Royal Children’s Hospital, Brisbane, QLD, Australia; 7 Arthur and Sonia Labatt Brain Tumour Research Centre, Program in Developmental and Stem Cell Biology, Hospital for Sick Children, University of Toronto, Toronto, ON, Canada

**Keywords:** Medulloblastoma subgroups, G-protein coupled receptors, Therapeutic targets, Imaging targets

## Abstract

**Background:**

Medulloblastoma is the most common malignant brain tumor in children. Genetic profiling has identified four principle tumor subgroups; each subgroup is characterized by different initiating mutations, genetic and clinical profiles, and prognoses. The two most well-defined subgroups are caused by overactive signaling in the WNT and SHH mitogenic pathways; less is understood about Groups 3 and 4 medulloblastoma. Identification of tumor subgroup using molecular classification is set to become an important component of medulloblastoma diagnosis and staging, and will likely guide therapeutic options. However, thus far, few druggable targets have emerged. G-protein coupled receptors (GPCRs) possess characteristics that make them ideal targets for molecular imaging and therapeutics; drugs targeting GPCRs account for 30-40% of all current pharmaceuticals. While expression patterns of many proteins in human medulloblastoma subgroups have been discerned, the expression pattern of GPCRs in medulloblastoma has not been investigated. We hypothesized that analysis of GPCR expression would identify clear subsets of medulloblastoma and suggest distinct GPCRs that might serve as molecular targets for both imaging and therapy.

**Results:**

Our study found that medulloblastoma tumors fall into distinct clusters based solely on GPCR expression patterns. Normal cerebellum clustered separately from the tumor samples. Further, two of the tumor clusters correspond with high fidelity to the WNT and SHH subgroups of medulloblastoma. Distinct over-expressed GPCRs emerge; for example, LGR5 and GPR64 are significantly and uniquely over-expressed in the WNT subgroup of tumors, while PTGER4 is over-expressed in the SHH subgroup. Uniquely under-expressed GPCRs were also observed. Our key findings were independently validated using a large international dataset.

**Conclusions:**

Our results identify GPCRs with potential to act as imaging and therapeutic targets. Elucidating tumorigenic pathways is a secondary benefit to identifying differential GPCR expression patterns in medulloblastoma tumors.

## Background

Medulloblastoma is an embryonal tumor of the cerebellum that accounts for 20% of all pediatric brain tumors and is the most common cause of death from CNS malignancy in children [1]. Furthermore, survivors face a multitude of long-term sequelae secondary to treatment; exposing a developing brain to the cytotoxic therapies that are currently offered can result in physical, neurological and intellectual disabilities [1-3]. Historically, medulloblastoma tumors have been treated according to a morphology-based classification system that divides tumors into three principle histopathologic classes: classic, desmoplastic/nodular and large cell/anaplastic (LCA) [4]. The histopathological class informs prognosis, for example tumors displaying LCA morphology generally have the worst prognosis [4]. However, recent advances have utilized genetic profiling to classify medulloblastoma tumors and these techniques have identified medulloblastoma subgroups that differ in both molecular and clinical profiles [5-7]. Various groups have identified between four and five potential subgroups [6,8-13]; however, a recent consensus conference determined that evidence supported four distinct subgroups and acknowledged the potential for multiple subtypes within each subgroup [7]. The two most well-defined subgroups are characterized by overactive signaling in the WNT and Sonic hedgehog (SHH) mitogenic pathways. Less is known about the underlying tumorigenesis mechanisms of the remaining two tumor subgroups, Group 3 and Group 4; however, specific genetic aberrations and gene expression characteristics have been found, and epigenetic origins to these tumors have been proposed [14-16]. These four principle medulloblastoma subgroups differ in terms of demographics, predominant histology, likely cell of origin, DNA copy number aberrations and molecular markers [5,7,17]. Importantly, the genetic profile has prognostic significance leading investigators to urge translation of genetic classification into clinical therapeutic trials [7,14,18,19]. Tumors of the WNT subgroup have the most favorable outcomes and SHH tumors have an intermediate response to current therapies. The recent development of small molecule inhibitors of the SHH pathway holds promise for the treatment of these tumor subgroups [20,21]. Group 3 tumors appear to have the worst prognosis using current therapeutic approaches [5]; however, Groups 3 and 4 are less well-characterized, both clinically and genetically, resulting in a lack of potential targets that has hindered the development of novel therapeutic strategies. Identification of tumor subgroup using molecular classification is expected to become an important component of medulloblastoma diagnosis and staging in the near future. Molecular classification will also likely be used to guide therapeutic options, to measure response to therapy and to provide early detection of relapse.

G-protein coupled receptors (GPCRs) are key regulators and points of control in both the SHH and WNT signal transduction pathways, as well as many other cell signaling mechanisms [22]. GPCRs possess characteristics that make them ideal targets for molecular imaging and therapeutics; including that they are membrane-bound, their ligands bind with high affinity and specificity, and that the receptor-ligand complex is subsequently endocytosed carrying the ligand into the tumor cell [23]. The utility of targeting GPCRs in medulloblastoma has been demonstrated with the advent of somatostatin receptor targeted imaging and therapy [24,25] and Octreoscans are now able to differentiate medulloblastoma from low-grade cerebellar tumors and scar tissue [25-28]. Molecularly targeted imaging has the potential to provide *in vivo* classification, and *in vivo* measurement of response to treatment as well as early detection of relapse. Furthermore, molecularly targeted chemo- or radiotherapy has the potential to decrease or alleviate long-term toxic effects of external beam radiotherapy.

While the molecular expression patterns of many genes and proteins in medulloblastoma subgroups have been discerned, subgroup-specific GPCR expression patterns have not previously been investigated. A subset of GPCRs appear on commonly used gene chips, such as the Affymetrix U133 chip; however these chips do not allow for the detection of under-expressed genes [29,30]. Our approach, using quantitative GPCR arrays (Taqman), allows for the assessment of both over- and under-expressed GPCRs.

The aim of this study was to discover G-protein coupled receptors that could serve as targets for imaging and therapeutic agents in medulloblastoma, and we have successfully identified potential receptor targets. Elucidating tumorigenic and potentiating mechanisms in medulloblastoma subtypes has been a secondary benefit to our study.

## Methods

### Human tumor cohort

Tumors analyzed for GPCR expression consisted of snap-frozen tumor tissues from 41 medulloblastomas, representing primary surgical resection tissue. Normal pediatric cerebellum was used as control tissue. Both specimen types were acquired from the Cooperative Human Tissue Network (Columbus, OH), The Queensland Children’s Tumour Bank (Queensland, AUS), The Children’s Cancer Research Unit at the Children’s Hospital at Westmead (Westmead, AUS), the Knight Cancer Institute Biolibrary at Oregon Health and Sciences University (Portland, OR) and from patients of the University of Iowa Hospitals and Clinics (UIHC) Children’s Hospital. Basic clinical data including age and sex were also obtained. The histopathological reports were acquired with the majority of tumor samples and more extensive pathology reports including cytogenetics were available for some patients. UIHC specimens were acquired under an Institutional Review Board (IRB) approval. Specimens acquired from other sources were de-identified and use of these tissues was declared “Not Human Research” by the University of Iowa IRB.

### RNA isolation and GPCR expression arrays

RNA was isolated from snap-frozen tumor tissue using the PerfectPure RNA Tissue Kit (5Prime); the quantity and quality of RNA was evaluated using a Nanodrop 1000 Spectrophotometer (Thermo Fischer Scientific) and an Agilent 2100 Bioanalyzer. RNA of sufficient quality was defined as having an RNA Integrity Number (RIN) of at least 6 on a scale of 1–10; RINs in the 8–9.5 range were most commonly seen. The High-Capacity Reverse Transcription Kit (Applied Biosystems) was used to convert the isolated RNA to cDNA. The resultant cDNA of each tumor sample was then applied to a TaqMan Human GPCR Array (Applied Biosystems) which contains 380 TaqMan Gene Expression Assays arranged in a 384 well–plate (four control wells are included). Each GPCR array was subsequently run on a 7900HT Fast Real-Time PCR System (Applied Biosystems) and the resulting data was analyzed using the SDS/Relative Quantification Manager v.1.2 and the DataAssist v.3.0 software packages (both Applied Biosystems).

### Statistical analysis

Statistical calculations were performed by the DataAssist (v3.0; Applied Biosystems) software. Maximum allowable C_T_ value was set at 40.0 and these values were included. The global normalization method was employed [31]. All p-values were adjusted using the Benjamin-Hochberg False Discovery Rate to correct for multiple testing and the occurrence of false positives. Heat maps are the result of unsupervised hierarchical clustering performed by DataAssist. Distances between tumor samples were calculated for clustering based on the ΔC_T_ values using Pearson’s Correlation; complete linkage was used as the clustering method.

### Histology

Formalin-fixed paraffin embedded (FFPE) tissues were obtained from the previously mentioned tissue banks in the form of 4 μm thick sections on slides. These tissues were routinely stained with hematoxylin and eosin (HE) to determine architectural and morphological features, including desmoplasia, nodular formation, and large-cell/anaplastic features. Dominant histologic category was determined by a neuropathologist.

### Immunohistochemistry

On cases in which FFPE material was available, subgrouping was accomplished following an immunohistochemical method established at St. Jude Children’s Research Hospital that uses immunoreactivity patterns to four antibodies (β-catenin, YAP1, GAB1 and filamin A) to categorize tumors into the WNT and SHH subgroups and Non-WNT/SHH tumors [32]. In this study, the SHH and WNT subgroups, and Non-SHH/WNT tumors were identified via immunoreactivity patterns to two of these markers: β-catenin (Abcam #ab16051) and YAP1 (Santa Cruz #sc-101199).

Antigen unmasking of paraffin sections was performed (citrate buffer, pH 6) in a decloaker and endogenous peroxidase activity was quenched with 3% hydrogen peroxide. Sections were incubated with the primary antibody (β-catenin at 1:2000; YAP1 at 1:1000) for 60 min (β-catenin) or 30 minutes (YAP-1) and then incubated with DAKO Mouse Envision HRP System reagent for 30 minutes for β-catenin or 15 minutes for YAP1. Slides were developed with DAKO DAB plus for 5 min followed by DAB Enhancer for 3 minutes before counterstaining with hematoxylin.

### Fluorescence in situ hybridization (FISH)

In cases in which there was sufficient material, FISH to determine *C-MYC* and/or *N-MYC* amplification was performed. The DNA probes *C-MYC* BAP and *N-MYC*/CEP2 (Abbott Molecular) were utilized. The sections with *C-MYC* copy number gains were sequentially probed with a CEP8 probe to further assess chromosome 8 copy number gains vs specific *C-MYC* amplification. The protocols for FFPE slide preparation and hybridization were as per manufacturer’s specifications. Briefly, after deparaffinizing, enzyme-pretreating and fixing the sections, the hybridizations were performed on a ThermoBrite (Abbott Molecular) programmed for melt temperature at 85°C and time for 2 minute. After overnight hybridization at 37°C, the slides were washed in 0.4XSSC/0.3% NP-40 for 2 minutes at 73°C and in 2XSSC/0.1% NP-40 for 1 minute at room temperature. The slides were then counterstained with DAPI. The slides were analyzed and images acquired using CytoVision computerized imaging system (Leica, USA).

### Independent correlation of GPCR expression patterns

Three independent previously published gene expression [8,10-12] datasets were analyzed using the R2 software (http://r2.amc.nl). Expression patterns of LGR5, GPR64, PTGER4, FZD2 and F2R were compared according to the four medulloblastoma subgroups. Differential expression of these candidate genes was assessed using one-way ANOVA. P-values < 0.05 were considered to be statistically significant.

## Results

### GPCR expression patterns

**Figure 1:**
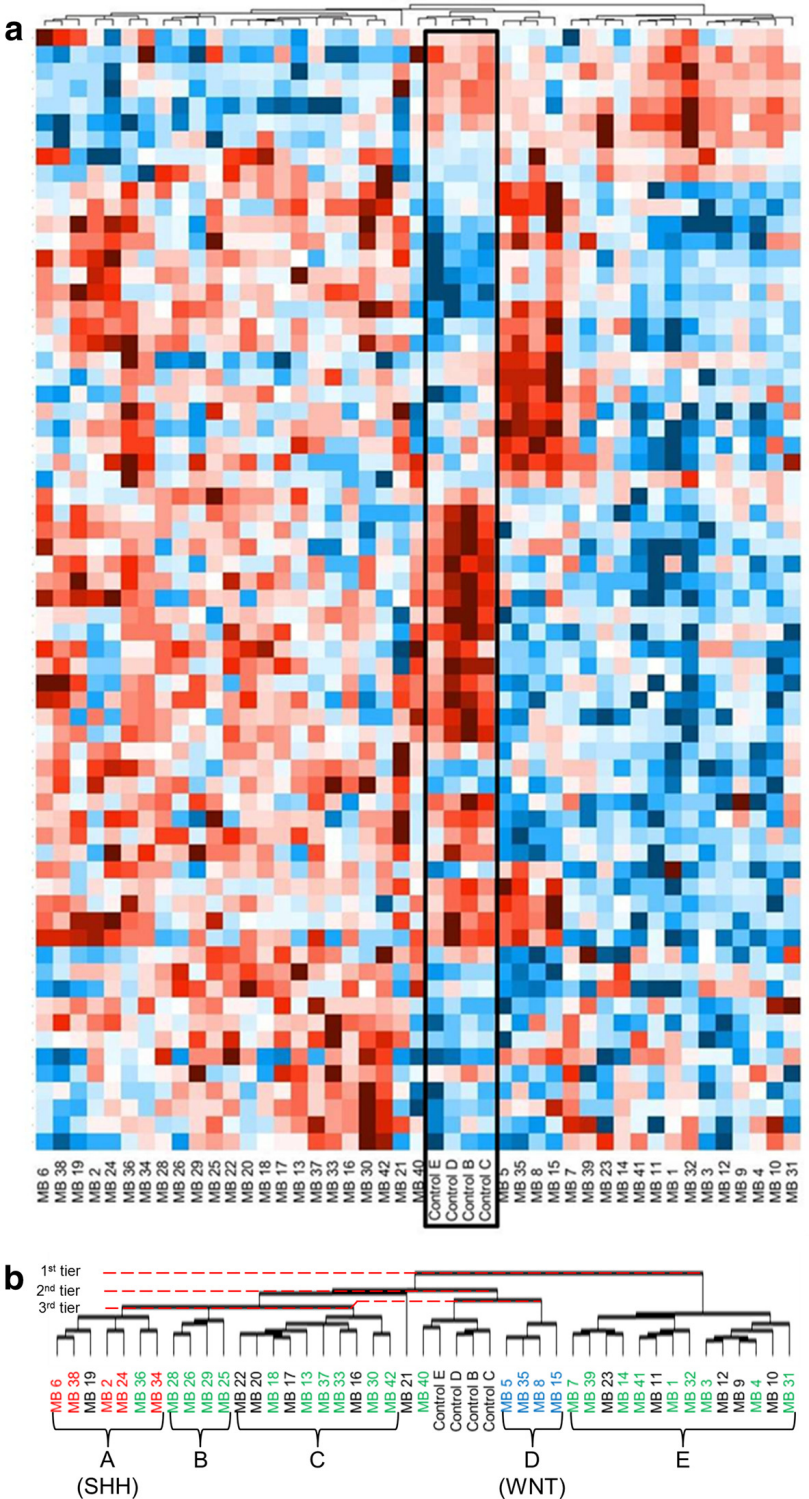
**GPCR expression patterns delineate distinct groups of medulloblastoma tumors.** The heat map represents GPCR expression levels in 41 medulloblastoma tumors compared to normal pediatric cerebella **(a)**. Red color indicates low C_T_ on qPCR, corresponding to high RNA expression; blue is high C_T_ and low RNA expression. Control cerebella (*Controls B, C, D, E)* are outlined in the black box. This heat map displays the results of unsupervised hierarchical clustering performed by DataAssist (v3.0; Applied Biosystems) software. Presented in this heat map are those GPCRs that were over- or under-expressed, as compared to control cerebellum, at a p ≤ 0.10 level. An enlarged image of the clustering stems, as well as the resultant tumor groups is seen in **(b)**. Distances between tumor samples were calculated for clustering based on the ΔC_T_ values using Pearson’s Correlation; complete linkage was used as the clustering method. Groups (*A-E*) of medulloblastoma tumors have emerged based solely on their GPCR expression patterns **(b)**. Subsequent immunohistochemical subtyping of the medulloblastoma samples identified tumors of the SHH, WNT and Non-WNT/SHH subgroups (Figure 2, Table 2). The SHH- and WNT-subgroup tumors clustered tightly together on the GPCR expression heat map. Red color indicates tumors identified as belonging to the SHH subgroup, blue indicates WNT subgroup tumors and green indicates tumors identified as Non-WNT/SHH. FFPE slides not available for classification for those medulloblastoma samples in black.

RNA from 41 human medulloblastoma tumors and four normal human cerebellum specimens were subjected to qPCR analysis of GPCR expression levels. Clusters of medulloblastoma tumors emerged based solely on their GPCR expression patterns (Figure 1; Additional file 1: Figure S1 includes the GPCR loci). Unsupervised hierarchical clustering of all 45 samples revealed varying numbers of groups, depending on the level of association. Two clusters of tissue samples emerged at the lowest level of association: one cluster of 14 tumors designated cluster *“E”* (Figure 1b and Table 1) and a second cluster including the remaining tumor 27 samples, as well as the four normal cerebellar controls.

**Table 1:** GPCR expression levels by linkage analysis clusters, compared to normal cerebella

	**Cluster “A”**		**Cluster “B”**		**Cluster “C”**		**Cluster “D”**		**Cluster E”**	
	**(n = 7)**		**(n = 4)**		**(n = 10)**		**(n = 4)**		**(n = 14)**	
	**Fold change**	**p-value**	**Fold change**	**p-value**	**Fold change**	**p-value**	**Fold change**	**p-value**	**Fold change**	**p-value**
FZD2	**25**	**0.01**	27	0.04	**16**	**0.01**	**45**	**0.01**	4.5	0.05
RPLP0	**15**	**0.01**	7.8	0.32	**9.8**	**0.01**	**28**	**0.01**	2.9	0.19
EDG4	**25**	**0.01**	22	0.04	**22**	**0.01**	20	0.03	7.8	0.04
PTGER4	**14**	**0.01**	2.0	0.43	**5.7**	**0.01**	2.9	0.30	1.1	0.90
GPR126	**17**	**0.01**	13	0.21	**15**	**0.01**	0.67	0.76	6.1	0.08
OR2C3	**0.035**	**0.01**	0.026	0.29	**0.0076**	**0.01**	0.12	0.49	1.1	0.89
FKSG83	**0.010**	**0.01**	0.037	0.22	0.30	0.26	0.61	0.69	2.7	0.31
F2R	**73**	**0.01**	54	0.04	55	0.02	29	0.03	15	0.03
DRD2	**75**	**0.01**	24	0.10	17	0.02	19	0.04	4.9	0.13
GPR142	0.078	0.60	**undetectable**	**5.E-03**	0.51	0.81	1.0	0.99	0.23	0.52
OPRM1	0.57	0.74	0.0028	0.22	**0.0063**	**0.01**	0.0032	0.23	**0.00050**	**3.E-03**
GPR147	0.059	0.15	0.011	0.29	**0.0056**	**0.01**	0.035	0.10	**0.00050**	**5.E-04**
GPR62	2.2	0.30	7.3	0.07	**14**	**0.01**	10	0.15	3.1	0.08
GPR153	3.3	0.10	8.7	0.21	**8.5**	**0.01**	0.50	0.24	3.0	0.04
PPYR1	0.59	0.63	0.033	0.21	**0.030**	**0.01**	0.47	0.37	1.2	0.82
EDG8	0.053	0.10	0.080	0.29	**0.11**	**0.01**	0.15	0.37	0.50	0.19
GPR160	2.4	0.19	5.9	0.22	**12**	**0.01**	1.2	0.90	1.4	0.61
SSTR3	0.40	0.61	0.13	0.49	**0.068**	**0.01**	0.72	0.62	0.99	0.99
GPR10	0.15	0.19	0.11	0.38	**0.082**	**0.01**	0.23	0.20	0.58	0.36
EDG7	0.42	0.48	0.12	0.56	0.12	0.03	**0.0079**	**0.01**	**0.032**	**1.E-03**
GRM6	19	0.22	326	0.29	248	0.02	**1539**	**0.01**	**79**	**0.01**
CCKBR	0.28	0.60	0.12	0.29	0.039	0.03	**0.0028**	**0.01**	**0.010**	**0.01**
OPRK1	0.29	0.49	1.0	1.0	0.21	0.20	**0.0027**	**0.01**	0.046	0.03
CHRM4	19	0.55	5.1	0.23	4.8	0.11	**74**	**0.01**	3.3	0.21
OPRD1	11.8	0.29	3.3	0.76	1.9	0.40	**344**	**0.01**	0.85	0.88
LGR5	0.50	0.71	0.83	0.95	0.45	0.55	**117**	**0.01**	0.22	0.17
GPR123	0.048	0.19	0.034	0.23	0.031	0.02	0.0041	0.15	**0.0016**	**4.E-04**
NTSR2	0.023	0.03	0.030	0.23	0.080	0.03	0.0030	0.04	**0.0056**	**5.E-04**
TRBV5	0.083	0.14	0.077	0.22	0.10	0.08	2.0	0.79	**0.0051**	**3.E-03**
ADORA1	0.83	0.91	0.28	0.32	0.14	0.14	3.0	0.33	**0.019**	**3.E-03**
ADRA1A	0.27	0.38	0.066	0.23	0.024	0.03	0.0082	0.19	**0.0088**	**0.01**
GRM4	0.13	0.30	0.11	0.32	0.037	0.06	0.0043	0.03	**0.0048**	**0.01**
HTR5A	0.074	0.17	0.12	0.29	0.17	0.15	0.0091	0.03	**0.017**	**0.01**
GPR84	0.20	0.19	0.39	0.54	0.10	0.05	0.045	0.04	**0.043**	**0.01**
GPR39	0.11	0.20	0.0068	0.05	0.015	0.02	0.0041	0.03	**0.0058**	**0.01**
GPR77	1.0	0.99	0.47	0.54	0.85	0.85	0.14	0.51	**0.041**	**0.01**
GPR37L1	0.049	0.19	0.057	0.22	0.080	0.11	0.0054	0.19	**0.0058**	**0.01**
GPR63	1.5	0.77	0.18	0.53	0.075	0.10	1.4	0.82	**0.012**	**0.01**
GPR75	0.48	0.43	0.15	0.21	0.22	0.11	0.13	0.06	**0.029**	**0.01**
OR2A4	0.51	0.50	5.7	0.22	3.7	0.08	2.1	0.51	**3.3**	**0.01**
CD97	4.6	0.12	1.4	0.68	1.8	0.11	1.5	0.67	**0.27**	**0.01**

* Fold-change <1 indicates decreased expression compared to normal cerebellum; all fold-change levels rounded to two significant digits. Significant fold-changes (p ≤ 0.01) indicated indicated in bold.

The next level of association split this cluster of 31 specimens (27 tumors and 4 controls) into two further clusters: 1) four tumor samples (cluster *“D”* in Figure 1b and Tables 1 and 2) four cerebellar control samples plus one tumor sample (Controls B, C, D, E and MB 40). The other 21 tumors could be further divided into three clusters designated *“C”* (n = 10), *“B”* (n = 4), and *“A”* (n = 7) in Figure 1b and Table 1. One tumor sample associated alone at this level (MB21). The cerebellar control samples display a GPCR expression profile that is very distinct from each of the five clusters of medulloblastoma tumors (clusters *“A,” “B,” “C,” “D”* and *“E;”* Figure 1).

**Table 2:** Immunohistochemical groupings of medulloblastoma tumors

		**β-catenin**	
		**Nuclear + Cytoplasmic**	**Cytoplasmic**
YAP1	Positive	MB5, MB8, MB15, MB35	MB2, MB6, MB24, MB34, MB38
		**WNT**	**SHH**
	Negative		MB1, MB3, MB4, MB7, MB13, MB14, MB18, MB25, MB26, MB27, MB28, MB29, MB30, MB31, MB32, MB33, MB36, MB37, MB39, MB40, MB41, MB42
			**Non-WNT/SHH**

* MB28 and MB37 displayed rare nuclei that were weakly positive for YAP1; both were considered negative for YAP1 immunoreactivity.

### GPCR expression levels in linkage analysis clusters

The fold-change in expression of GPCRs between tumor and normal tissue was evaluated in the distinct clusters (*A-E*) of medulloblastoma. Table 1 summarizes the GPCRs that were over- or under-expressed at a significant level (p≤ 0.01) in one or more clusters compared to normal cerebella. No GPCRs were significantly altered in all five clusters at this significance level.

Among the 380 GPCRs probed, nine GPCRs displayed significantly altered expression in cluster *“A;”* seven were over-expressed, ranging from 14-fold (PTGER4) to 75-fold (DRD2) expression, while two (OR2C3 and FKSG83) were under-expressed, compared to normal cerebella (Table 1).

One GPCR (GPR142) exhibited significantly altered expression in cluster *“B;”* GPR142 expression was undetectable in this cluster. There were no significant alterations in expression levels of GPR142 in the other clusters, compared with normal cerebella.

Expression of 15 GPCRs was significantly altered in cluster *“C;”* six of these GPCRs were common between clusters *“A”* and *“C”* and two other GPCRs were common between clusters *“C”* and *“E.”* In cluster *“C,”* over-expression was seen in eight of the GPCRs, ranging from 5.7-fold (PTGER4) to 22-fold (EDG4) expression; under-expression in seven GPCRs ranged from 0.01-fold (OR2C3, OPRM1 and GPR147) to 0.11-fold (EDG8) compared to normal cerebellum (Table 1).

Nine GPCRs displayed significantly altered expression levels in cluster *“D;”* two of these GPCRs were common to both clusters *“A”* and *“C”* while three other GPCRs were common to cluster *“E”* (Table 1). Six of the nine GPCRs with altered expression levels in cluster *“D”* exhibited over-expression, ranging from 28-fold (RPLP0) to 1500-fold (GRM6).

Twenty GPCRs had significantly altered expression in cluster “*E”* (Table 1). Two of these GPCRs were common to cluster *“C”* and three were common to cluster *“D.”* Of the 20 GPCRs with altered expression levels in cluster *“E,”* only two were over-expressed (GRM6 and OR2A4) while the other 18 were under-expressed, as compared to normal cerebella.

### Immunohistochemical analysis and categorization

Formalin-fixed, paraffin-embedded (FFPE) blocks of tumor tissue were available for thirty of the tumors that had been assayed for GPCR expression levels. Immunoreactivity was determined by two independent University of Iowa pathologists, with any differences being resolved between two readers. Additionally, sections of medulloblastoma tumor samples obtained through the Queensland Children’s Tumour Bank (MB30 – MB41) were separately probed for immunoreactivity to the same antibodies at Pathology Queensland (Australia). These sections were read by an independent Pathology Queensland pathologist; therefore, for these samples (MB30- MB41), there are three independent readers. A high level of agreement was observed between the two different laboratories.

Tumors were classified based on immunoreactivity patterns, as shown in Table 2. Immunoreactivity to YAP1 has been shown to differentiate WNT and SHH tumors from Non-WNT/SHH (Groups 3 and 4) tumors [32]. Immunoreactivity to YAP1 was found in nine out of 31 tumors (Table 2). Nuclear immunoreactivity to β-catenin is a well-established method for the identification of WNT – driven medulloblastoma tumors [5,32]. Nuclear β-catenin staining in less than 2% of tumor nuclei was considered sporadic and these samples were read as negative for nuclear β-catenin staining [32]. Four tumor samples displayed nuclear β-catenin staining. All four of these tumors positive for nuclear β-catenin also displayed YAP1 immunoreactivity, and have therefore been classified as a WNT subtype medulloblastoma (Table 2).

**Figure 2:**
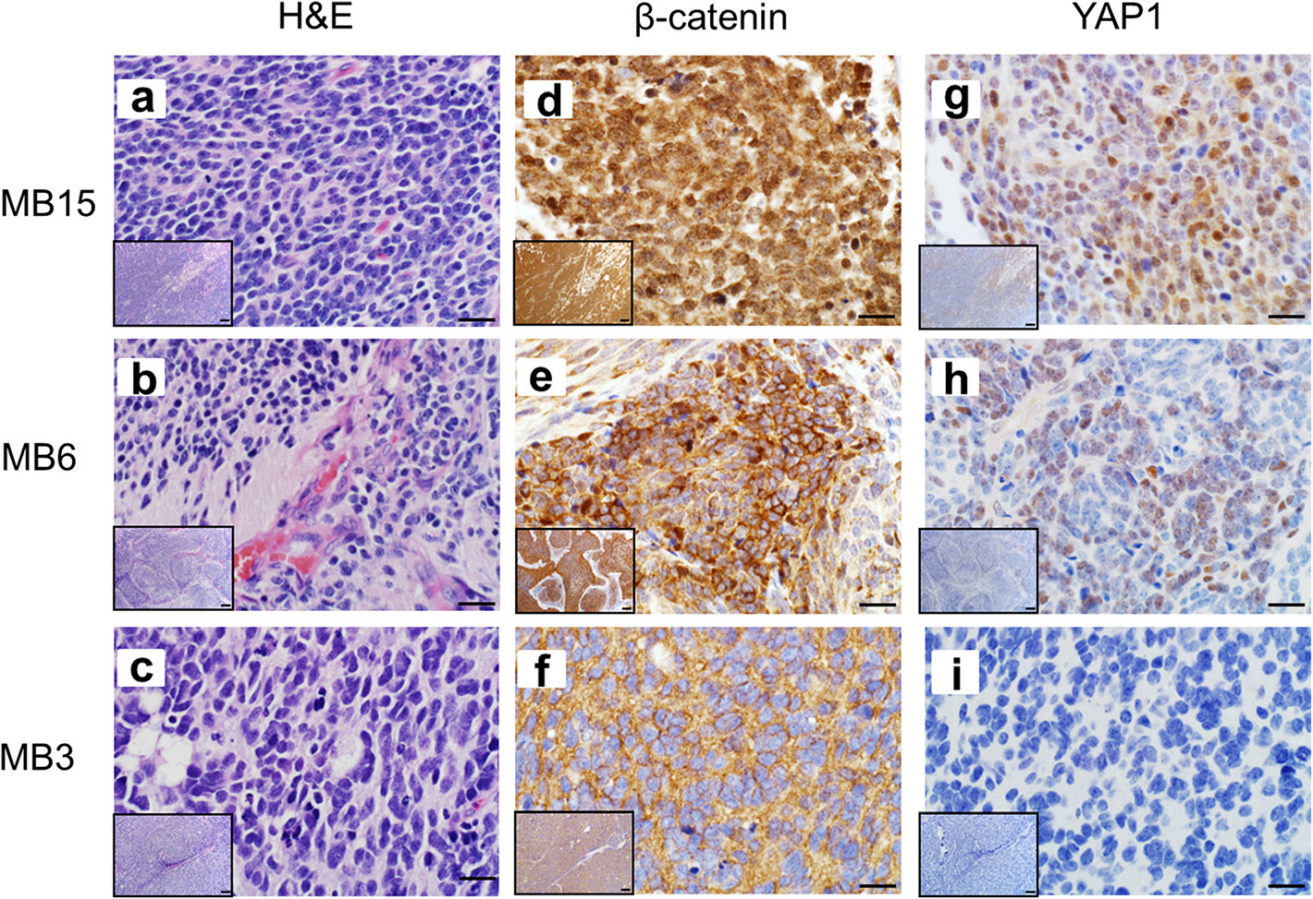
**Examples of immunohistochemical (IHC) staining in human medulloblastoma tumors.** A method for differentiation of molecular medulloblastoma subgroups using immunoreactivity to four markers was establish by Ellison DW et al. in 2011 [32]. The H&E, β-catenin and YAP1 staining of three representative medulloblastoma tumor samples is shown in panels **a** – **i**. MB 15 **(a)** displays classical morphology, MB 6 **(b)** is an example of desmoplastic morphology and includes extensive focal nodularity, and MB 3 **(c)** displays anaplastic characteristics. MB 15 demonstrated nuclear and cytoplasmic immunoreactivity to β-catenin **(d)** and was positive for YAP1 staining **(g)**, characterizing it as belonging to the WNT molecular subgroup. Conversely, MB 6 displayed only cytoplasmic β-catenin staining **(e)**, and was positive for YAP1 **(h)**, indicating that it is of the SHH subgroup. Lastly, MB 3 is cytoplasmically immunoreactive for β-catenin **(f)** and negative for YAP1 **(i)** staining, thus placing this tumor in the Non-WNT/SHH category. Staining for the above markers has been evaluated in 30/41 medulloblastoma tumors. The scale bars in panels a - i represent 20 μm; the scale bar in the inset images in each panel represents 100 μm.

Combining the findings from the immunoreactivity patterns to YAP1 and β-catenin provides a method of differentiating the WNT, SHH and non-WNT/SHH subgroups of tumors. A combination of YAP1 immunoreactivity and nuclear β-catenin staining (Figure 2d, h) segregated the WNT subgroup (n = 4; 13%), as shown in Table 2. Positive YAP1 staining without nuclear β-catenin staining (Figure 2e, i) indicated the SHH subgroup (n = 5; 17%); non-WNT/SHH subgroups were characterized by a lack of immunoreactivity to both of these antibodies (Figure 2f, i; n = 22; 70%). The remaining 10 tumors were not classified due to lack of FFPE tissue for the performance of immunohistochemical analysis. Our observed distribution of tumors into the subgroups closely aligns with previously published distributions in larger cohorts [5].

### Medulloblastoma subgroups

#### WNT pathway medulloblastomas

The WNT pathway medulloblastomas (n = 4) were identified by a combination of positive YAP1 staining, as well as nuclear and cytoplasmic immunoreactivity to β-catenin (Figure 2, Table 2). All WNT tumors displayed classic histopathology, characterized by sheets of monomorphic cells with hyperchromatic nuclei and a high nuclear: cytoplasmic ratio (Figure 2) [4]. C-MYC and N-MYC amplification was probed using fluorescent in-situ hybridization (FISH); the N-MYC signal was normal in all four WNT subgroup tumors and no C-MYC amplification was observed, though two WNT tumors displayed increased C-MYC signal due to gains of chromosome 8 (Additional file 2: Table S1). Of the four WNT tumors, 50% were from male patients, and the age range for all tumors was 5 to 17 years.

The WNT tumors (MB5, MB8, MB15, MB35) tightly clustered together (Figure 1, Table 2) and completely correlate to linkage analysis cluster *“D”* (Figure 1b). Aside from each other, the WNT tumors were most closely associated with the normal cerebellar control samples. To determine GPCR expression patterns specifically in this subgroup, the WNT tumors were grouped together and the fold-change in expression level of each receptor, as compared to normal controls, was assessed.

The expression levels of 26 GPCRs, out of the 380 receptors probed, were significantly (p ≤ 0.05) altered in WNT tumors compared to expression levels in normal cerebella (Additional file 3: Table S2). Of these 26 GPCRs, 12 were expressed at a significantly lower level than in normal cerebella, while 14 were over-expressed (Additional file 3: Table S2). The levels of under-expression ranged from 0.003-fold (OPRK1) to 0.07-fold (RHO), while the levels of over-expression ranged from 8.8-fold (MRGPRE) to 2200-fold (GPR64). Four of the over-expressed GPCRs within the WNT subgroup (FZD2, F2R, EDG4 and RPLP0) were also over-expressed to a significant level within the SHH subgroup tumors and the Non-WNT/SHH tumors. Five GPCRs (EDG7, CCKBR, GRM4, NTSR2 and GPR84) were significantly under-expressed in the WNT subgroup and Non-WNT/SHH tumors, while GRM6 and DRD2 were significantly over-expressed in both groups.

#### SHH pathway medulloblastomas

Positive immunoreactivity to YAP1, combined with non-nuclear β-catenin staining identified tumors of the SHH subgroup (n = 5; Figure 2, MB6; Table 2). One SHH subgroup tumor displayed classic histopathology, two tumors exhibited desmoplasia with nodularity and one tumor had anaplastic features. One tumor was classified as having complex histopathology with multiple morphological features. C-MYC and N-MYC FISH data were available for four of the five SHH tumors; all four of these tumors displayed normal C-MYC and N-MYC signals (Additional file 2: Table S1). Within the SHH subgroup, two patients were female, two were male; age and gender of the patient were unknown for one tumor. Ages of the patients ranged from 1.0 to 2.5 years.

The SHH-subgroup of tumors corresponds to the linkage analysis cluster *“A”* established by GPCR expression patterns (Figure 1). All five SHH subgroup tumors (MB2, MB6, MB24, MB34 and MB38) clustered together in a grouping of seven tumor samples; one Non-WNT/SHH tumor sample (MB36) and one tumor sample for which FFPE tissue was not available for categorization (MB19) also clustered in this group (Figure 1).

Seven GPCRs in the SHH subgroup displayed significantly altered expression levels when compared to normal cerebellum (p ≤ 0.05; Additional file 3: Table S2). Six of these altered GPCRs demonstrated over-expression, ranging from 14-fold (RPLP0) to 72-fold (F2R) expression, while one (FKSG83) displayed under-expression (0.01-fold). As discussed above, four GPCRs were also over-expressed to a significant level within all three (WNT, SHH, Non-WNT/SHH) categorized tumor groups (FZD2, RPLP0, EDG4 and F2R). There were no GPCRs that were significantly altered and common to both the SHH and WNT, but not the other subgroups. One GPCR (GPR126) was altered exclusively in the SHH and the Non-WNT/SHH subgroups and two GPCRs (PTGER4 and FKSG83) were uniquely altered only in the SHH subgroup tumors.

#### Non-WNT/SHH medulloblastomas

A lack of YAP1 immunoreactivity in medulloblastoma tumors is indicative of the Non-WNT/SHH subgroup (encompassing both Groups 3 and 4 [32]). Twenty-two tumors were negative for YAP1 (Figure 2, MB3; Table 2). Moreover, all 22 of these tumors also lacked nuclear β-catenin immunoreactivity (Figure 2f), as would be expected for Non-WNT/SHH tumors. Of these 22 tumors, 10 displayed purely classic histopathology, four tumors had classic histopathology along with areas demonstrating anaplastic features, three tumors displayed desmoplastic or nodular/desmoplastic qualities and five tumors exhibited purely large-cell, anaplastic or anaplastic morphology (Additional file 2: Table S1). C-MYC and N-MYC FISH was performed in 18 Non-WNT/SHH subgroup tumors; high-level amplification of C-MYC was seen in three Non-WNT/SHH tumors (MB25, MB31 and MB39) while six tumors displayed increased C-MYC copy numbers due to gains of chromosome 8, and nine tumors had normal C-MYC signal (Additional file 2: Table S1). N-MYC amplification was seen in three Non-WNT/SHH tumors, MB4, MB37 and MB40 (N-MYC/CEP2 ratio of 1.88, 1.8 and 2.3, respectively) and four tumors displayed increased N-MYC signal due to gain of chromosome 2 (Additional file 2: Table S1). Patient characteristics were available for 15 of these tumors; 11 out of 15 tumors were from male patients and patient ages ranged from 1.9 to 8.4 years.

A broad viewing of the GPCR expression patterns heat map shows that the Non- WNT/SHH tumors reside in two large tumor groups, interspersed with tumors for which immunohistochemistry -based subgroup categorization was not possible (Figure 1b). The lowest level of association clusters together 14 tumor samples; this cluster of 14 tumor samples corresponds to the GPCR expression patterns linkage analysis cluster *“E.”* Nine of these tumors are of the Non-WNT/SHH subgroup (MB1, MB3, MB4, MB7, MB14, MB31, MB32, MB39, and MB41) and the remaining five tumors in this cluster were uncategorized (MB9, MB10, MB12, MB11 and MB23), as shown in Figure 1b.

Association clusters “*B*” and “*C*” encompass the majority of the remaining non-WNT/SHH tumors (Figure 1b). One Non-WNT/SHH group tumor (MB40) clustered with the normal control cerebella samples, and one Non-WNT/SHH group tumor (MB36) clustered with the SHH subgroup tumors (Figure 1b). As discussed above, within one of the second tier clusters, three third tier clusters emerged (n = 10, 4, 7); the third tier cluster of four tumors was comprised entirely of Non-WNT/SHH tumors (MB25, MB26, MB28, MB29) and corresponds to linkage analysis cluster *“B”* (Figure 1b and Table 1), while the third tier cluster of ten tumors was comprised of six Non-WNT/SHH tumors (MB13, MB15, MB30, MB33, MB37 and MB42) and four immunohistochemically uncategorized tumors (MB15, MB17, MB20, MB22) and correlates to cluster *“C”*. GPCR expression data was not available for one Non-WNT/SHH subgroup tumor (MB27) due to insufficient quality of mRNA.

When comparing GPCR expression levels between the Non-WNT/SHH subgroup tumors and the normal control cerebella, 31 GPCRs displayed significantly altered expression levels (p ≤ 0.05; Additional file 3: Table S2). Twelve of these GPCRs were over-expressed in Non-WNT/SHH tumors compared to control; level of over-expression ranged from 2.8-fold (OR2A4) to 164-fold (TACR3). The level of under-expression in 19 of the 31 altered GPCRs ranged from 0.0018-fold (GPR147) to 0.23-fold (EDG8).

### Independent correlation of GPCR expression patterns

**Figure 3:**
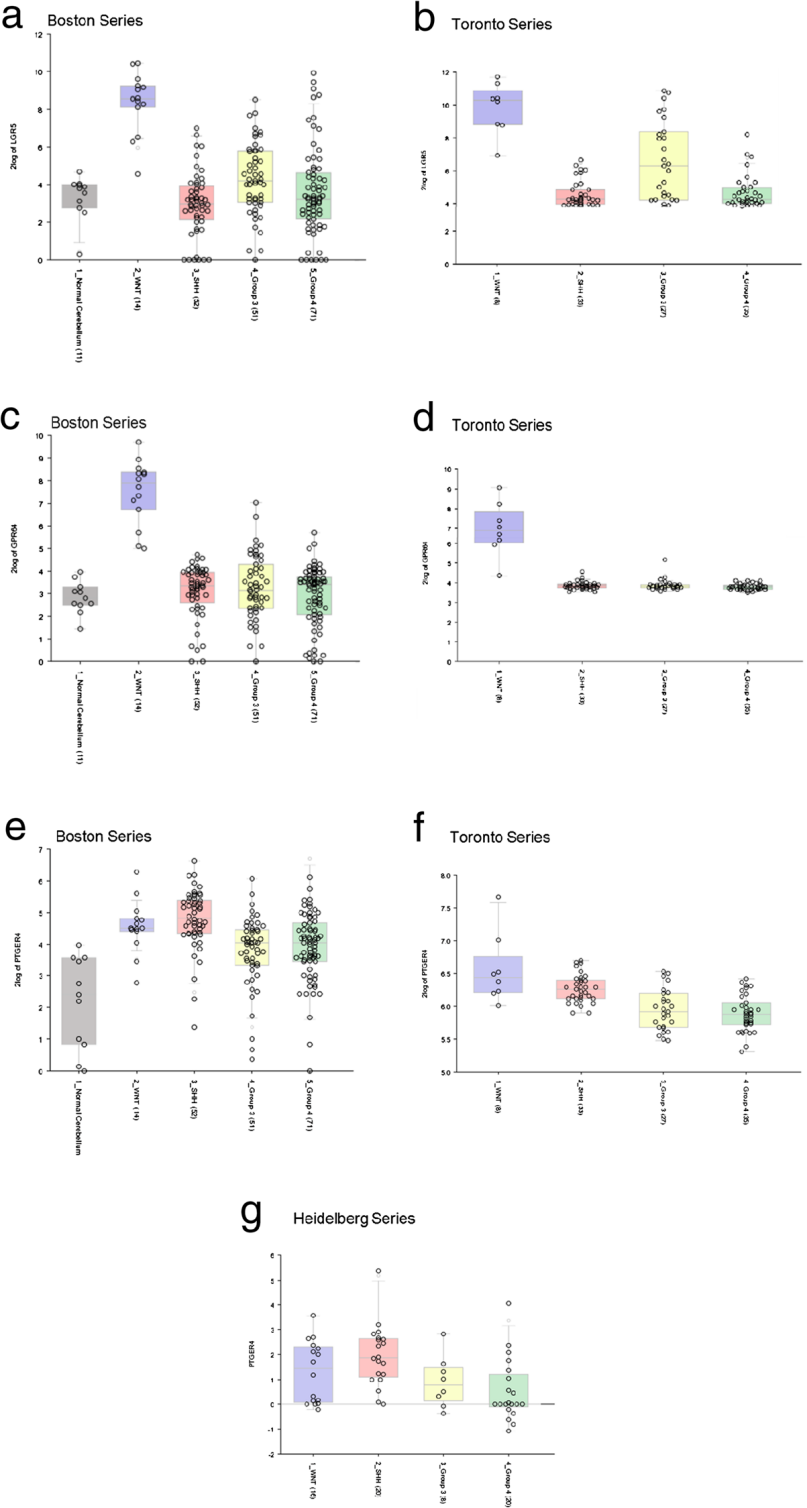
**Gene expression profiling data from large cohorts of medulloblastoma tumors qualitatively support our results.** In our data set, LGR5 was uniquely over-expressed (120-fold, p =0.01) in the WNT subgroup of tumors compared to normal cerebellum. Over-expression of LGR5 in WNT tumors has been demonstrated in two previously published sets of gene profiling data **(a, b)**[8,10]. Our data demonstrate over-expression of GPR64 in the WNT subgroup of tumors (2200-fold, p = 0.04). This aberration was also reported for WNT tumors in the Boston and Toronto series **(c, d)**[8,10]. PTGER4 was uniquely over-expressed in the SHH subgroup of tumors in our data set (16-fold, p = 0.02). This pattern of expression was also observed in the Boston and Heidelberg series **(e, g)**[8,11]; in the Toronto series **(f)**[10], the SHH subgroup shows increased PTGER4 expression as compared to Group 3 and Group 4 tumors, but not compared to the WNT subgroup. Blue boxes represent WNT tumors, red boxes represent SHH tumors, yellow boxes represent Group 3 tumors and green boxes represent Group 4 tumors **(a, b, c, d, e, f, g)**. In the Boston series **(a, c, e)**, the grey boxes represent normal cerebellar controls.

**Figure 4:**
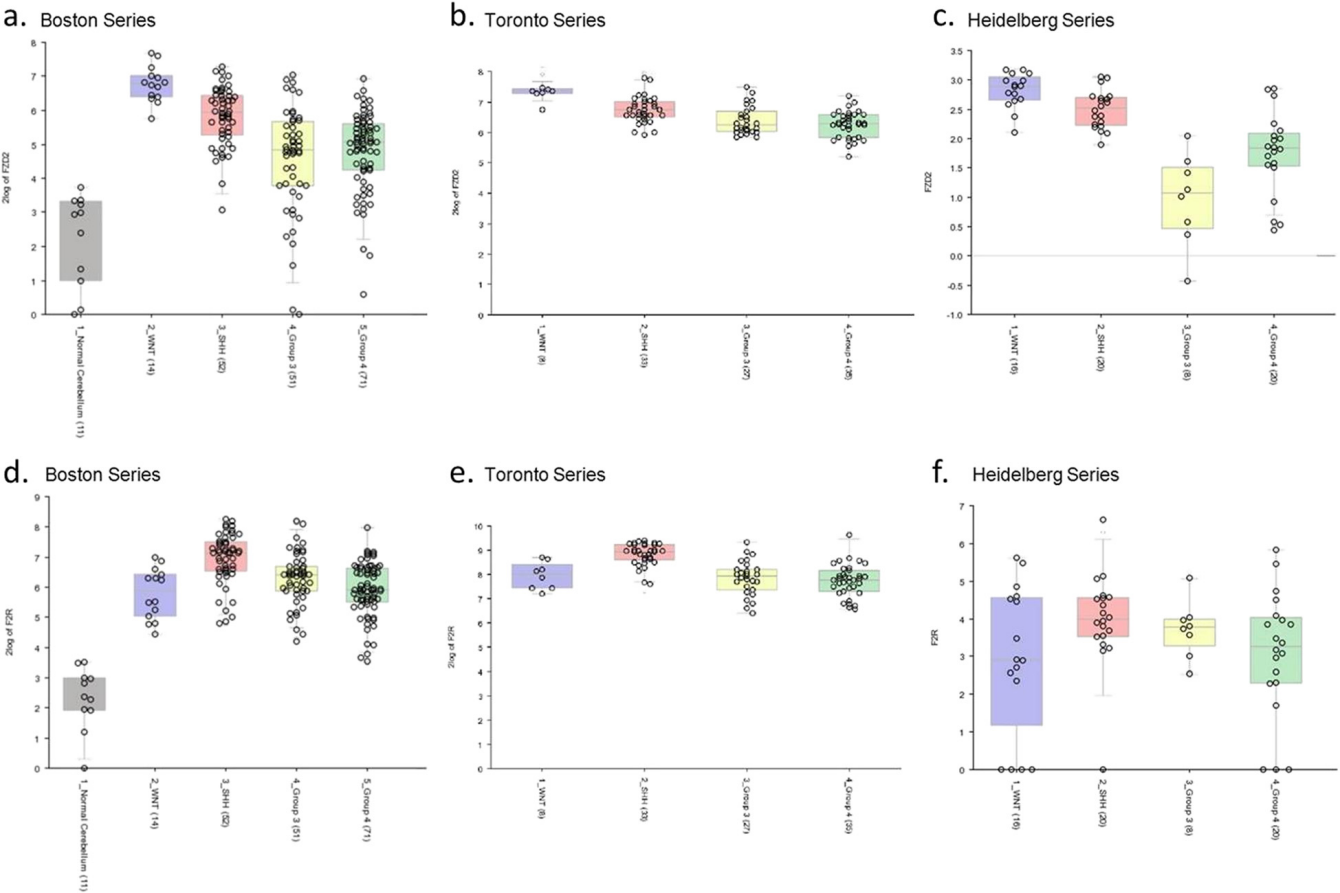
**Gene expression profiling data from large cohorts of medulloblastoma tumors qualitatively support our results.** Both FZD2 and F2R were significantly over-expressed in all subgroups of medulloblastoma tumors in our cohort (Additional file 3: Table S2). Three previously published sets of gene profiling data [8,10,11] found the same pattern to be true for both FZD2 **(a, b, c)** and F2R **(d, e, f)**. Blue boxes represent WNT tumors, red boxes represent SHH tumors, yellow boxes represent Group 3 tumors and green boxes represent Group 4 tumors **(a, b, c, d, e, f)**. In the Boston series **(a, d)**, the grey boxes present normal cerebellar controls.

Analysis of previously published gene expression profiling data from three independent cohorts of medulloblastoma patients (Toronto, Boston and Heidelberg series [8,10,11], respectively), clearly demonstrates that the GPCR expression patterns observed in our data set hold consistent in the larger cohorts. For example, in two cohorts of subtyped medulloblastoma tumors (188 medulloblastomas and 11 control cerebella [8], and 103 medulloblastomas [10]), both LGR5 and GPR64 were found to be expressed at higher levels in the WNT subgroup tumors, as compared to both normal cerebella and the other three subgroups of tumors (Figure 3a-d); these expression characteristics support our results (Additional file 3: Table S2). Furthermore, we found PTGER4 to be uniquely over-expressed in the SHH subgroup of medulloblastoma (16-fold, p = 0.02; Additional file 3: Table S2) and this finding replicated what was also seen in the Boston and Heidelberg tumor cohorts (Figure 3e, g) [8,11]. PTGER4 expression in the SHH subgroup was increased in comparison to its expression in Group 3 and Group 4 tumors (t-probabilities = 1.6e^-6^, 2.7e^-8^, respectively), but not WNT tumors, in the Toronto series of medulloblastoma patients (Figure 3f) [10]. Likewise, F2R and FZD2, both of which were found to be significantly over-expressed in all subgroups of medulloblastoma tumors in our dataset (Additional file 3: Table S2), were also both highly expressed in all tumor groups in previously published larger tumor cohorts (Figure 4) [8,10,11].

## Discussion

The primary goal of this project was to identify G-protein coupled receptors that could serve as targets for imaging and therapeutic agents in medulloblastoma, and this has been successful. GPCR expression patterns also have the potential to elucidate initiating and proliferative mechanisms. The results of this study indicate that GPCR expression patterns delineate five groups of medulloblastoma tumors, two of which correlate with high fidelity to the WNT and the SHH subgroups of medulloblastoma [5,7,10,14,17,18,32]. Distinct GPCRs are uniquely over-expressed in the WNT and SHH subgroups, as well as in three other groups of tumors, strongly suggesting that GPCR targets specific to each medulloblastoma subgroup can be identified. Additionally, these data indicate that the unique GPCR expression patterns found may help clarify important mechanistic differences between the groups.

Development of new, or utilization of known ligands to uniquely over-expressed GPCRs offer the potential to provide patient specific information. Subgroup targeted imaging, using radiolabeled GPCR ligands, would offer a non-invasive method to simultaneously diagnose medulloblastoma and characterize molecular subgroups. Importantly, targeted imaging would also afford a sensitive technique for follow-up imaging to determine response to therapy and the presence of metastatic lesions. A benefit in developing imaging agents targeting over-expressed receptors is that the downstream action of the targeted receptor need not correspond to specific tumorigenesis mechanisms. Rather, the viability of an imaging agent is dependent on the following criteria: high affinity and specificity for the target receptor, reliably and highly differential expression of the target receptor between tumor and normal tissue, limited off-target or off-tissue effects, and size constraints. In regard to medulloblastoma, the ability to cross the blood–brain barrier is also crucial. The utility of such a GPCR-targeted imaging modality has already been proven: The Octreoscan, which employs SPECT imaging to detect radiolabeled somatostatin receptor analogues has the ability to differentiate medulloblastoma from low-grade cerebellar tumors and provides an imaging modality to differentiate recurrent medulloblastoma from scar tissue, as well as to localize metastatic lesions [24,25,28]. Positron emission tomography (PET) imaging provides a higher degree of sensitivity than SPECT imaging [28] and somatostatin receptor targeted agents are being adapted for use with PET imaging [28]. Recent evidence has shown that high expression of somatostatin receptors, particularly in non-SHH subgroup tumors, is correlated to an increased survival and may have potential as a prognostic marker [36]. However, previous studies report that somatostatin receptor expression is uniformly high in all medulloblastoma tumors [37,38]. Though these studies were performed prior to the advent of medulloblastoma subgrouping, they indicate that somatostatin receptor targeted agents will fail to distinguish between the subgroups of medulloblastoma. Somatostatin receptor, type 2, expression trends towards over-expression in all subgroups, however does not reach significance in our current data set (WNT subgroup: 7.6-fold, p = 0.24; SHH subgroup: 5.1-fold, p = 0.34; Non-WNT/SHH: 8.1-fold, p = 0.23).

GPCR-targeted therapeutics, either radioablative or chemically based, have the potential to reduce the need for external beam radiation, and the highly toxic effects associated with cranial spinal radiation treatment [2]. Therapeutically, GPCR antagonists represent the most instinctive approach to counteracting the proliferative signals transduced by some GPCRs within the context of cancer [39]. As such, GPCR antagonists have previously been investigated as potential chemotherapeutic targets in a variety of malignancies [39]. Our data indicate that drugs targeting specific GPCRs may not display the same efficacy in all medulloblastoma tumors, and that subgroup specific GPCR targets would likely result in more beneficial outcomes. GPCR targeted radiotherapy, in which an isotope, such as lutetium-177 or yttrium-90, is attached to a receptor ligand, antibody or other molecule in order to target the radioactivity to the desired GPCR, has been successful in the treatment of neuroendocrine tumors and other malignancies [40,41].

While each of the differentially expressed GPCRs in our data set deserves active investigation into its potential as a target, several candidate receptors are outlined below. Twenty-six GPCRs exhibited significantly altered expression levels in the WNT subgroup tumors; thirteen of these were unique to the WNT subgroup, and eight of these thirteen displayed overexpression, as compared to normal cerebella, and thus are candidate receptors for targeting. Leucine-rich repeat containing G-protein-coupled receptor 5 (LGR5) is significantly over-expressed in the WNT subgroup of tumors (120-fold, p =0.01); additionally, our data suggest that it is mildly under-expressed in other groups of medulloblastoma, providing the benefit of being highly differentially expressed not only between WNT tumors and normal cerebella, but also between WNT tumors and other medulloblastoma groups (Additional file 3: Table S2; Figure 3). Furthermore, LGR5 holds the intuitive advantage of being involved in the WNT signaling pathway; LGR5, a known marker of certain adult stem cells, forms a complex with Frizzled/LRP and acts to potentiate the WNT signal [42]. The role of LGR5 in cancer biology has been well-described, especially in the realm of gastrointestinal cancers, where antibodies to LGR5 can be used to identify colorectal cancer stem cells [43,44]. Recently, R-spondins, a class of four large, secreted proteins known to enrich WNT signaling, have been identified as high affinity ligands for LGR5 (and LGR4) [45]. R-spondin proteins hold limited potential as imaging or therapeutic agents in medulloblastoma due to their large size (35 kDa) [46] and likely inability to cross the blood–brain barrier [47,48]. However, the emergence of high-throughput screening facilities provides the resources to potentially identify key binding elements of the R-spondin proteins. Our data suggest that the development of small-molecule agents targeting the LGR5 receptor is worthy of attention.

GPR64 displays a favorable differential expression profile for a WNT subgroup target (2200-fold, p = 0.04; Additional file 3: Table S2, Figure 3). GPR64 is an orphan receptor that belongs to a family of adhesion proteins and is normally highly expressed only in the epididymis; however it has recently been found to be expressed in Ewing’s sarcoma (ES), as well as other carcinomas, and represents a marker of invasiveness and metastatic potential in ES [49]. The exact signaling mechanism that follows GPR64 activation is yet unknown and a direct connection between GPR64 and WNT signaling is not readily apparent; however, the development of imaging and radiotherapeutic targets is not dependent on the role of downstream mechanisms in proliferation or apoptosis. Due to its differential expression in medulloblastomas, as well as the fact that it is normally only expressed in the epididymis, GPR64 represents a promising candidate for the development of imaging or radiotherapeutic agents that could be potentially efficacious not only in WNT subgroup medulloblastomas, but also Ewing’s sarcoma.

PTGER4 is a GPCR that was uniquely over-expressed (16-fold, p = 0.02) in the SHH group of tumors (Additional file 3: Table S2). It was also over-expressed in “Cluster C” GPCR-grouped medulloblastomas (5.66-fold, p = 0.01; Table 1), however these tumors fell into the Non-WNT/SHH subgroup and the same pattern of PTGER4 expression was not seen in that subgroup as a whole. PTGER4, or EP_4_, is a receptor for prostaglandin E_2_ (PGE_2_). PGE_2_ has been shown to act as a growth promoting molecule that stimulates proliferation, angiogenesis and invasion [50], and is present at high levels in a variety of malignancies [51,52]. Furthermore, the role of PGE_2_, and its receptors, has been investigated within the context of medulloblastoma [53]. PGE_2_ induces medulloblastoma cell proliferation *in vitro*, while inhibition of PGE_2_ activity was suppressive both *in vitro* and *in vivo*[53]. While Baryawno and colleagues [53] found that PGE_2_ receptors EP_1-3_ were most important in stimulating medulloblastoma cell growth, our data suggest that tumor subgrouping may affect PGE_2_’s role. Small molecule antagonists to EP_4_ are currently in development for the treatment of inflammatory pain [54]; EP_4_ represents a particularly viable therapeutic target, as blockage at this site does not interfere with the production of other important prostanoids, and thus avoids the cardiovascular side effects that can be seen with blockage of this pathway [54]. EP_4_ represents a viable potential target in medulloblastoma, a possibility that is furthered by the fact that inhibition of the prostaglandin cascade has been shown to enhance the cytotoxic effects of radiotherapy [35] presenting the possibility of synergistic combination therapy. Interestingly, PGE_2_ has been shown to potentiate the WNT signaling cascade, both in colorectal cancer cells [34], as well as in normal adult hematopoietic stem cells [55], and it was recently found that PGE_2_ upregulates LGR5 [43]. This finding highlights the important crosstalk between the WNT and SHH signaling cascades [56].

While over-expressed GPCRs provide potential targets, their under-expressed counterparts are equally pertinent when probing unanswered mechanistic questions. GPCRs are responsible for initiating intracellular signaling for multiple pathways; these receptors act at the cell surface to integrate and coordinate diverse communicative stimuli between cells, and converge on shared downstream modulators and effectors. Identifying GPCRs down-regulated in medulloblastoma subgroups may pinpoint receptors critical for growth suppression or inhibition, whose under-expression can lead to, or potentiate, the development of cancer. Less is known about the initiating mechanisms at play in Groups 3 and 4 medulloblastomas [7]; identifying differentially under-expressed GPCRs may help identify additional pathways that contribute to tumorigenesis in these subgroups. MTNR1A, a GPCR for melatonin, is significantly under-expressed only in the Non-WNT/SHH group of medulloblastoma tumors (0.0065-fold, p = 0.02). Melatonin has been postulated to be a tumor suppressor gene due to its oncostatic effect in various cancers [33,57,58], as such, expression of MTNR1A was found to be frequently silenced through methylation of CpG islands surrounding the MTNR1A promoter in cases of oral squamous cell carcinoma (OSCC) and other primary cancers [59]. Furthermore, forced expression of MTNR1A in cells led to growth suppression, suggesting that loss of MTNR1A activity plays a role in the pathogenesis of OSCC [59]; similar results have also been found in breast cancer cell lines [60] and in prostate epithelial cells [61]. The anti-proliferative effect observed in prostate epithelial cells was demonstrated to be due to MTNR1A-mediated activation of protein kinase A (PKA) and protein kinase C (PKC) with a subsequent increase in p27 (kip1) gene transcription. The p27 gene encodes for cyclin-dependent kinase inhibitor 1B, a protein that prevents the activation of cyclin E-CDK2 or cyclin D-CDK4 complexes, thus regulating cell cycle progression. A similar mechanism may be at play in Non-WNT/SHH medulloblastomas.

Another GPCR, the adenosine A_1_ receptor (ADORA1) has a known role in growth suppression [62,63]. In colon cancer cells, adenosine, via ADORA1, induces apoptosis by activating caspases [63]. Additionally, it has been reported that deletion of ADORA1 leads to an increase in glioblastoma tumor growth, however this observed effect was believed to be mediated through tumor-adjacent microglia [64]. ADORA1 was under-expressed in the Non-WNT/SHH group (0.088-fold, p = 0.03), again suggesting that loss of ADORA1 activity may play a role in the pathogenesis of a Non-WNT/SHH medulloblastoma tumors, especially those seen in Cluster “E” (Table 1).

Our data identify GPCRs whose expression is significantly altered in subgroups of medulloblastoma; while many of these alterations reach significant levels, a limitation of our study was the restricted sample size available. To partially alleviate this concern, we worked with the Medulloblastoma Advanced Genomics International Consortium (MAGIC), an international consortium that aims to stratify and characterize medulloblastoma through genomics. Our key findings, specifically the over-expression of LGR5 and GPR64 in the WNT subgroup tumors and F2R and FZD2 in all medulloblastoma, were mirrored in three independent international cohorts of subgrouped medulloblastoma (Figures 3 and 4). Though our data cannot be quantitatively combined with these larger data sets, a qualitative comparison adds substantial confidence and weight to our results.

## Conclusions

In summary, this study has shown that GPCR expression patterns differentiate the WNT and SHH subgroup of tumors. We have identified under-expressed GPCRs that may aid in discerning additional tumor- initiating, or potentiating, pathways at play in medulloblastoma. And importantly, we have pinpointed uniquely over-expressed GPCRs that hold potential as both imaging and therapeutic targets in the WNT and SHH medulloblastoma subgroups.

## Competing interests

The authors declare that they have no conflict of interests.

## Authors’ contributions

KLW and MSO co-conceived and led the study. KLW planned and executed the GPCR expression arrays and analyses, aided in the IHC analyses, performed the over-all analyses and drafted the manuscript; EAB performed initial GPCR expression arrays; KNGC developed IHC protocols and provided IHC analysis; PAK provided IHC analysis; BWD provided FISH analysis; QQ performed FISH studies and aided in FISH analysis; WJI provided biospecimens (including RNA) that formed a portion of our medulloblastoma cohort, as well as valuable input regarding interpretation of results and manuscript preparation; TR performed and provided IHC analyses on a subset of our medulloblastoma cohort; MR and MDT provided comparison studies on the expression of select GPCRs in large international cohorts; MR provided valuable input regarding interpretation of results and manuscript preparation; MSO provided financial and technical infrastructure, oversaw the study and significantly aided in drafting the manuscript. All authors read and approved the final manuscript.

## Supplementary Material

Additional file 1: Figure S1 GPCR expression patterns delineate distinct groups of medulloblastoma tumors. The heat map represents GPCR expression levels in 41 medulloblastoma tumors compared to normal cerebella. This heat map is the same as is seen in Figure 1, with GPCR names included along the y-axis. Control cerebella are outlined in the black box. Click here for file

Additional file 2: Table S1 Additional medulloblastoma tumor characteristics. Click here for file

Additional file 3: T3able S2 GPCR expression levels by subgroup, compared to normal cerebella. Click here for file
