# Supplementary material for: G-protein coupled receptor expression patterns delineate medulloblastoma subgroups
Source: Acta Neuropathol Commun. 2013 Oct 10;1:66. doi: 10.1186/2051-5960-1-66 (PMC3893540; doi:10.1186/2051-5960-1-66)
Supplement: Additional file 1: Figure S1 — GPCR expression patterns delineate distinct groups of medulloblastoma tumors. The heat map represents GPCR expression levels in 41 medulloblastoma tumors compared to normal cerebella. This heat map is the same as is seen in Figure 1, with GPCR names included along the y-axis. Control cerebella are outlined in the black box. [file 2051-5960-1-66-S1.docx]

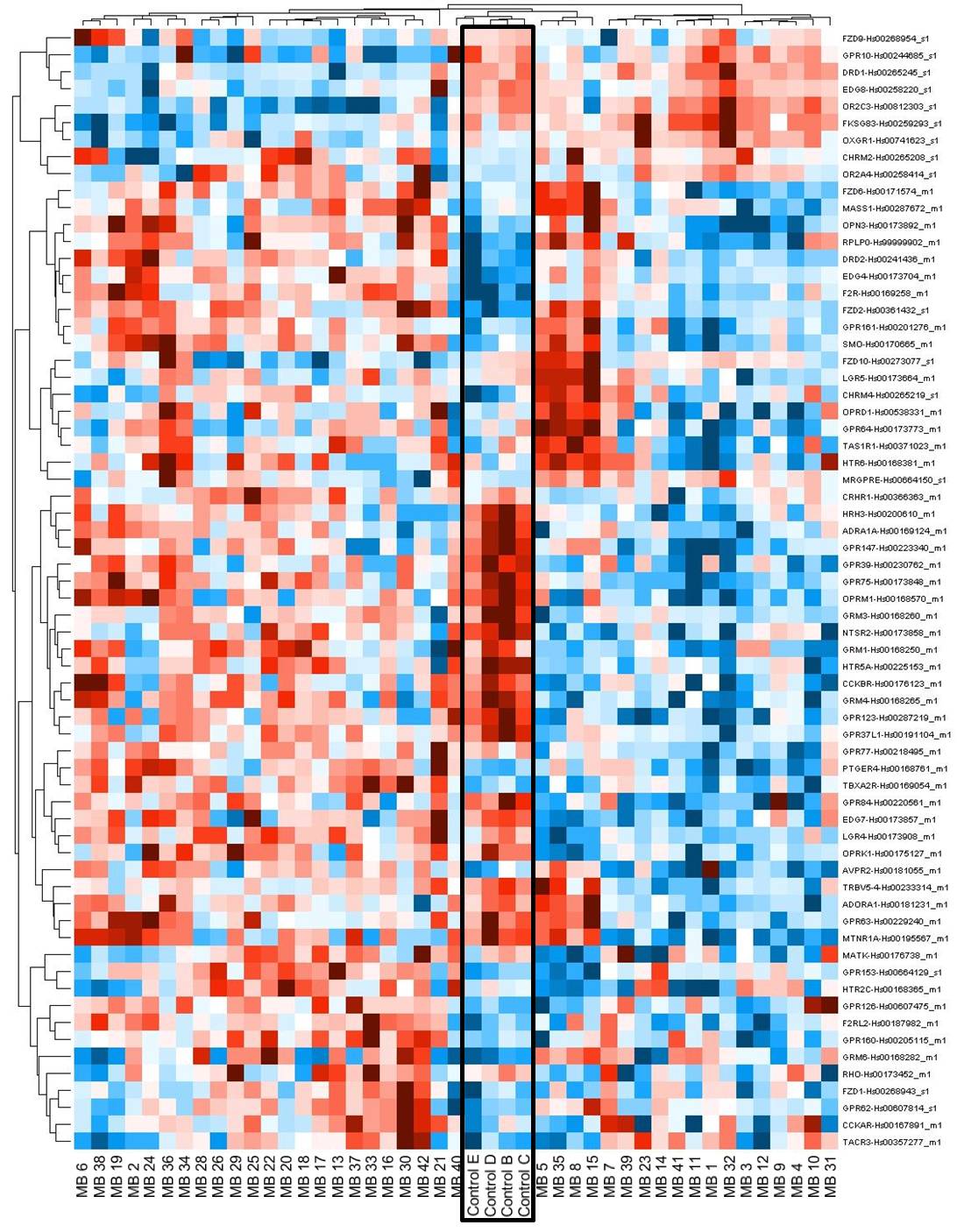
**Fig. S1** GPCR expression patterns delineate distinct groups of medulloblastoma tumors. The heat map represents GPCR expression levels in 41 medulloblastoma tumors compared to normal cerebella. This heat map is the same as is seen in Fig 1, with GPCR names included along the y-axis. Control cerebella are outlined in the black box.

“G-protein coupled receptor expression patterns delineate medulloblastoma subgroups.”

*Acta Neuropathologica Communications*

Kelsey L Whittier, Erin A Boese, Katherine N Gibson-Corley, Patricia A Kirby, Benjamin W Darbro, Qining Qian, Wendy J Ingram, Thomas Robertson, Marc Remke, Michael D Taylor, M Sue O’Dorisio*.*

Corresponding author: M Sue O’Dorisio, University of Iowa; sue-odorisio@uiowa.edu
