# Supplementary material for: G-protein coupled receptor expression patterns delineate medulloblastoma subgroups
Source: Acta Neuropathol Commun. 2013 Oct 10;1:66. doi: 10.1186/2051-5960-1-66 (PMC3893540; doi:10.1186/2051-5960-1-66)
Supplement: Additional file 2: Table S1 — Additional medulloblastoma tumor characteristics. [file 2051-5960-1-66-S2.docx]

**Table S1** Additional medulloblastoma tumor characteristics

* **AMP** = amplified
