# Supplementary material for: G-protein coupled receptor expression patterns delineate medulloblastoma subgroups
Source: Acta Neuropathol Commun. 2013 Oct 10;1:66. doi: 10.1186/2051-5960-1-66 (PMC3893540; doi:10.1186/2051-5960-1-66)
Supplement: Additional file 3: T3able S2 — GPCR expression levels by subgroup, compared to normal cerebella. [file 2051-5960-1-66-S3.docx]

**Table S2** GPCR expression levels by subgroup, compared to normal cerebella

* Fold-change <1 indicates decreased expression compared to normal cerebellum; all fold-change levels rounded to two significant digits. Significant fold-changes (p < 0.01) indicated with red (over-expression) and blue (under-expression) shading.
